# Single-cell RNA-seq analysis reveals the role of PGRN in modulating fibroblast heterogeneity and enhancing wound healing

**DOI:** 10.3389/fbioe.2026.1930259

**Published:** 2026-10-13

**Authors:** Wenjiang Yin, Chaohang Chen, Qixin Chen, Xulong Huang, Zhong Zhou, Chunming Zhao, Xingru Liu, Jingyuan Ma, Zhouru Li, Shanshan Li

**Affiliations:** 1 Department of Forensic Medicine, Xuzhou Medical University, Xuzhou, Jiangsu, China; 2 Jiangsu Medical Engineering Research Center of Gene Detection, Xuzhou Medical University, Xuzhou, Jiangsu, China; 3 The First Clinical Medical College, National Demonstration Center for Experimental Basic Medical Science Education, Xuzhou Medical University, Xuzhou, Jiangsu, China; 4 Department of Human Anatomy, Xuzhou Medical University, Xuzhou, Jiangsu, China

**Keywords:** angiogenesis, fibroblast, heterogeneity, progranulin, single-cell RNA sequencing, skin wound healing

## Abstract

**Introduction:**

The wound microenvironment plays a crucial role in the skin wound healing process. Fibroblasts, one of the critical cell types within this microenvironment, exhibit significant heterogeneity that influences healing outcomes. Previous studies have demonstrated that progranulin (PGRN) may reduce fibrogenesis during wound healing. However, the underlying mechanisms remain poorly understood. Thus, this study aims to investigate the specific effects of PGRN on fibroblast heterogeneity of skin wound healing in mice.

**Methods:**

To assess the role of PGRN, BALB/c mice were subcutaneously injected with PGRN overexpression or control lentivirus. Excisional skin surgery was performed a week later on the dorsal skin of the mice. Wound healing was monitored up to 21 days post-wounding, and specimens were collected at 7 days post-wounding for single-cell RNA sequencing. Bioinformatics analysis included cell clustering, marker gene identification, GO/KEGG enrichment, pseudotime trajectory, and cell-cell communication. Spatial transcriptomic data were used to analyze the spatial expression of genes of interest. Multi-immunohistochemical staining, western blot, and laser speckle blood flow imaging were performed to validate key molecular findings.

**Results:**

Our analysis revealed 33 distinct cell clusters, with a notable increase in the proportions of macrophages, neutrophils, NK cells, and myofibroblasts in the PGRN overexpression group, while fibroblasts and epithelial cells were less represented. Importantly, PGRN overexpression specifically stimulated the proliferation of myofibroblast subcluster 2 and induced unidentified fibroblast subclusters 0 and 5 that were enriched in pathways related to extracellular matrix and angiogenesis. Subcluster 0 fibroblasts were further characterized using the markers Crabp1, Prss35, Fabp5, and Acta2. Their location in the upper layer of the neodermis in the wound was confirmed by spatial transcriptomic data and multi-immunohistochemical staining. Additionally, PGRN treatment significantly enhanced blood flow in newly formed tissue and accelerated wound closure.

**Conclusion:**

Our findings indicated that PGRN regulated wound healing by promoting specific fibroblast populations, particularly those associated with angiogenesis and extracellular matrix remodeling. This study provides valuable insights into the complex regulatory mechanisms of PGRN in wound healing and highlights its potential as a therapeutic target for enhancing skin regeneration in clinical settings.

## Introduction

Wound healing is essential for patient recovery and quality of life, making it a significant focus of clinical research. Scarring, a common consequence of wound healing, may negatively impact both physical and mental health, resulting in functional limitations and a sense of inferiority. Research in this field is especially valuable for trauma and plastic surgery, which aims not only to restore skin integrity but also to minimize scarring and improve aesthetic outcomes. This process requires a delicate balance between tissue regeneration and fibrogenesis, highlighting the complexity of skin repair mechanisms. Understanding the regulatory mechanisms of skin wound healing is crucial for developing innovative therapies to improve healing and reduce scarring (Monavarian et al., 2019; Amjadian et al., 2022).

Progranulin (PGRN), a multifunctional growth factor, has attracted attention because of its involvement in diverse biological processes, such as cell proliferation, inflammation, tissue repair, and fibrosis (Yang et al., 2024). In intact healthy skin, PGRN is slightly expressed in keratinocytes. However, the PGRN level significantly increases and remains elevated for more than 20 days post-wounding (dpw), comprising the inflammatory and proliferative phases of wound healing. During this period, PGRN can be detected in various cell types, including endothelial cells, neutrophils, monocytes, macrophages, and fibroblasts (He et al., 2003; Li et al., 2019).

During the early stages of wound healing, PGRN is hydrolyzed by elastase into 6 kDa granulins (GRNs), which stimulate the production of proinflammatory cytokines. During the repair phase, secretory leukocyte protease inhibitor (SLPI) forms a complex with PGRN, thereby preventing elastase from converting PGRN into GRNs. Intact PGRN may bind to tumor necrosis factor receptors (TNFRs) and block the TNFα/TNFR complex, exerting anti-inflammatory effects (Zhu et al., 2002; Liu and Bosch, 2012). PGRN also plays a role in regulating fibrosis by inhibiting the phosphorylation of nuclear transcription factor κB (NF-κB), which provides protective effects against liver damage, inflammation, and fibrosis (Yoo et al., 2019). Our previous reports revealed that PGRN silencing exacerbates fibrosis after injury, whereas PGRN overexpression effectively reduces fibrosis (Li et al., 2019; 2023). PGRN exerts its antifibrotic effects by binding to and upregulating heat shock protein 40 family member C3 (DNAJC3) and inhibiting the SRF/CTGF/αSMA signaling pathway (Li et al., 2023). Moreover, PGRN enhances corneal wound healing by accelerating corneal re-epithelialization and neuronal regeneration (Yan et al., 2024).

Given that PGRN plays a significant role in skin wound healing, this study aims to elucidate the specific cellular heterogeneity and functional implications of PGRN treatment in mouse skin wounds through single-cell RNA sequencing (scRNA-seq), thereby providing novel insights into its therapeutic potential in enhancing wound healing and decreasing fibrosis. These results suggest that PGRN exerts a significant regulatory effect on fibroblasts and other cells in the wound microenvironment.

Fibroblasts are recognized as one of the most critical cell types within the wound microenvironment and directly influence fibrogenesis. They participate in extracellular matrix component synthesis, wound edge contraction, and inflammatory responses, thereby regulating tissue repair and remodeling, which makes them essential targets for improving wound healing and organ fibrosis (Weiskirchen et al., 2019; Talbott et al., 2022). Recent studies have highlighted the heterogeneity of fibroblast populations, which exhibit remarkable diversity in terms of their origin, phenotype, and function. Dermal fibroblasts are categorized into various subtypes, including dermal papilla, dermal sheath, papillary, reticular, arrector pili muscle, preadipocyte, adipocyte, pericyte, and fascia fibroblasts (Ganier et al., 2022). Papillary fibroblasts are known for their antiscarring properties, whereas reticular fibroblasts may promote scar formation (Xue et al., 2022).

Myofibroblasts, activated forms of fibroblasts, have attracted attention in the areas of tissue repair and fibrosis because of their unique characteristics and functions. Recent studies have highlighted the heterogeneity within myofibroblast populations, suggesting that they are not a uniform cell type but instead comprise various subpopulations with distinct phenotypic and functional attributes (Gao et al., 2024). Since the diversity of fibroblast and myofibroblast populations highlights the complexity of tissue repair, determining the regulatory mechanisms is important.

This study examined the role of PGRN in the cellular and molecular processes of wound healing. Our findings could not only enhance the understanding of wound healing mechanisms but also contribute to the development of innovative strategies for treating fibrotic diseases and improving patient outcomes in clinical practice.

## Materials and methods

### Animal experiments

34 Five-week-old male BALB/c mice were purchased from Cyagen Biosciences, Inc (Guangzhou, China). This study was approved by the Ethics Committee of Xuzhou Medical University (Xuzhou, Jiangsu Province, China). After 1 week of adaptive feeding, the dorsal skin of the mice was randomly injected subcutaneously with PGRN-overexpressing lentivirus (PGRN‒OE) or negative control lentivirus (NC) (1 × 10^7^/mouse) (Hunan Fenghui Biotechnology Co., Ltd., Hunan, China). One week later, skin excisional surgery was performed on both sides of the mouse dorsal skin according to previous methods. Prior to surgery, all mice received 1.25% tribromoethanol (Avertin) at 0.2 mL/10 g body weight via intraperitoneal injection. For analgesia, meloxicam was administered subcutaneously at 4 mg/kg before surgery, followed by once daily subcutaneous injections at the same dose for 3 consecutive days. The excision site was disinfected pre- and postoperatively. After surgery, mice were placed on a heated recovery pad until fully awake. Wound healing was monitored at scheduled intervals. Additional supportive care included provision of a palatable nutritional gel and veterinary attention as required. All mice wound images at 3, 7, 10, 14, and 21 dpw were taken and analyzed by ImageJ (version 1.54). The mice were euthanized by intraperitoneal injection of sodium pentobarbital at 150 mg/kg, and the skin around the wound (1 cm × 1 cm) was collected for subsequent experiments at 7, 14, and 21 dpw (5 mice/day/group).

### Tissue processing and single-cell sequencing

Four samples of 7dpw (2 PGRN‒OE and 2 NC) were washed with cold phosphate-buffered saline (PBS) and minced into small pieces on ice separately. After being enzymatically digested with collagenase I (Worthington) and DNase I (Worthington) for 45 min at 37 °C with agitation, the samples were sieved through a 70 μm cell strainer. The pelleted cells were suspended, the red blood cells were removed, and the cells were refiltered through a 40 μm cell strainer. The single-cell suspensions were further enriched with a MACS dead cell removal kit (Miltenyi Biotec). The number of cells was counted, and the viability of the cells was assessed with calcein-AM (Thermo Fisher Scientific) and Draq7 (BD Biosciences). The cell suspension was loaded into Chromium microfluidic chips with 3′ chemistry (v2) and barcoded via a 10×Chromium Controller (10X Genomics) by Shanghai Genechem Co., Ltd. RNA extracted from the barcoded cells was subsequently reverse-transcribed, and sequencing libraries were constructed via reagents from a Chromium Next GEM Single Cell 3’ (v2) reagent kit (10X Genomics) according to the manufacturer’s instructions. Sequencing was performed with an Illumina instrument (NovaSeq 6,000), depending on the project), according to the manufacturer’s protocols (Illumina).

### Data analysis

Single-cell sequencing data were deposited in the NCBI database (https://www.ncbi.nlm.nih.gov) under project number PRJNA1332166. The analysis of single-cell sequencing data was performed by Shanghai Genechem Co., Ltd. Fastp was used to perform basic statistics on the initial quality control (QC) of the raw reads. A gene with expression in more than 3 cells was considered expressed, and each cell was required to have at least 200 expressed genes. The raw reads were demultiplexed and mapped to the reference genome via the 10X Genomics Cell Ranger pipeline (https://support.10xgenomics.com/single-cell-gene expression/software/pipelines/latest/what-is-cell-ranger) via default parameters. The sequencing results are presented in the gene expression matrix, and the subsequent analyses were all performed on this basis. Uniform manifold approximation and projection (UMAP) was performed for cell cluster identification. Highly variable genes for each cluster were selected, and the validated skin cell marker genes were used to annotate the cell type. These single-cell analyses were performed via Cell Ranger and Seurat, with P values < 0.01 and fold changes >2 (Macosko et al., 2015; Satija et al., 2015). The cells were reclustered via Python 3.12.7. Additionally, proportion analysis and marker gene annotation were performed. Gene Ontology (GO) enrichment and KEGG pathway analyses of the marker genes were performed via the clusterProfiler R package. Monocle3 was used for pseudotime analysis, and CellPhoneDB was used for cell‒cell communication.

The GSE178758 spatial transcriptomic data were analyzed via CROST (https://ngdc.cncb.ac.cn/crost/home) (Wang et al., 2024) with the default settings. Specifically, a sample (VISDS000185) of *Mus musculus* skin at 7 dpw was obtained, and the locations of Crabp1, Prss35, Fabp5, and Acta2 were visualized. The spatial transcriptome data, along with our single-cell sequencing data, were subjected to integrated analysis via R, during which the spatial transcriptome data were reannotated.

### Tissue preparation and staining

The skin samples were fixed with 4% paraformaldehyde (Biosharp, Guangzhou, China), embedded in paraffin following standard protocols, and then cut into 3 µm thick slices. The slices were stained with hematoxylin and eosin (H&E, hematoxylin, Foruca, United States; eosin, Shanghai Chenyun Chemical Co. Ltd., China). Multi-immunohistochemical staining was performed via a series of staining cycles: after rehydration, antigen retrieval, blocking, and the application of primary and corresponding secondary antibodies, the slices were incubated with a tyramide fluorescence solution (APEXBIO, Texas, United States) for 10 min. Antigen retrieval was performed after each staining cycle to eliminate any antibody-TSA complexes. Nuclei were labeled with Hoechst 33,258 (KeyGEN BioTECH, Jiangsu, China). A confocal laser scanning microscope (CLSM; Leica STELLARIS 5, Germany) was used for slide visualization. The specifications of the antibodies used are as follows: anti–PRSS35 (GeneTex, GTX123037, Taiwan, China), anti–CRABP1 (Proteintech, 12588-1-AP, Hubei, China), anti-FABP5 (Santa Cruz Biotechnology, sc-365236, MA, United States), and anti-Acta2 (Proteintech, 67735-1-Ig, Hubei, China).

### Protein preparation and immunoblotting

The skin samples were chopped and ground with a glass homogenizer on ice. The samples were subsequently mixed with precooled lysis buffer (Beyotime Institute of Biotechnology, Shanghai, China) supplemented with a 1% protease inhibitor ‘cocktail’ (Vicmed, Xuzhou, Jiangsu, China). The homogenate was centrifuged twice at 12,000 × g for 15 min at 4 °C, and the resulting supernatant was collected. Protein quantification was performed via the BCA method (Beyotime Institute of Biotechnology, Shanghai, China). Protein samples were separated via SDS‒PAGE and transferred to PVDF membranes. After blocking with 5% skim milk, the membranes were incubated with the following indicated antibodies: anti–PRSS35 (GeneTex, GTX123037, Taiwan, China), anti–CRABP1 (Proteintech, 12588-1-AP, Hubei, China), and anti–GAPDH (Santa Cruz Biotechnology, sc365062, MA, United States). All the blots were developed with enhanced chemilumine scence (ECL) (Biosharp, BL520B, Anhui, China).

### Skin blood flow measurement

After hair removal, a laser speckle blood flow imager (SIM BFI H R PRO, Hubei, China) was utilized to monitor the blood flow of the wounded skin and surrounding area. The instrument was set to default settings. For each wound, five consecutive blood flow images were recorded at 1-s intervals.

## Results

### Effect of PGRN on skin wound healing

We first assessed the role of PGRN in wound healing rates. Observations of the wounds revealed that, compared with the NC group, the PGRN‒OE group had a reduced wound area, indicating that PGRN contributed to wound closure (Figure 1A). Morphological analysis revealed a reduced fibrotic area in the PGRN‒OE group compared with the NC group (Figure 1B).

**FIGURE 1:**
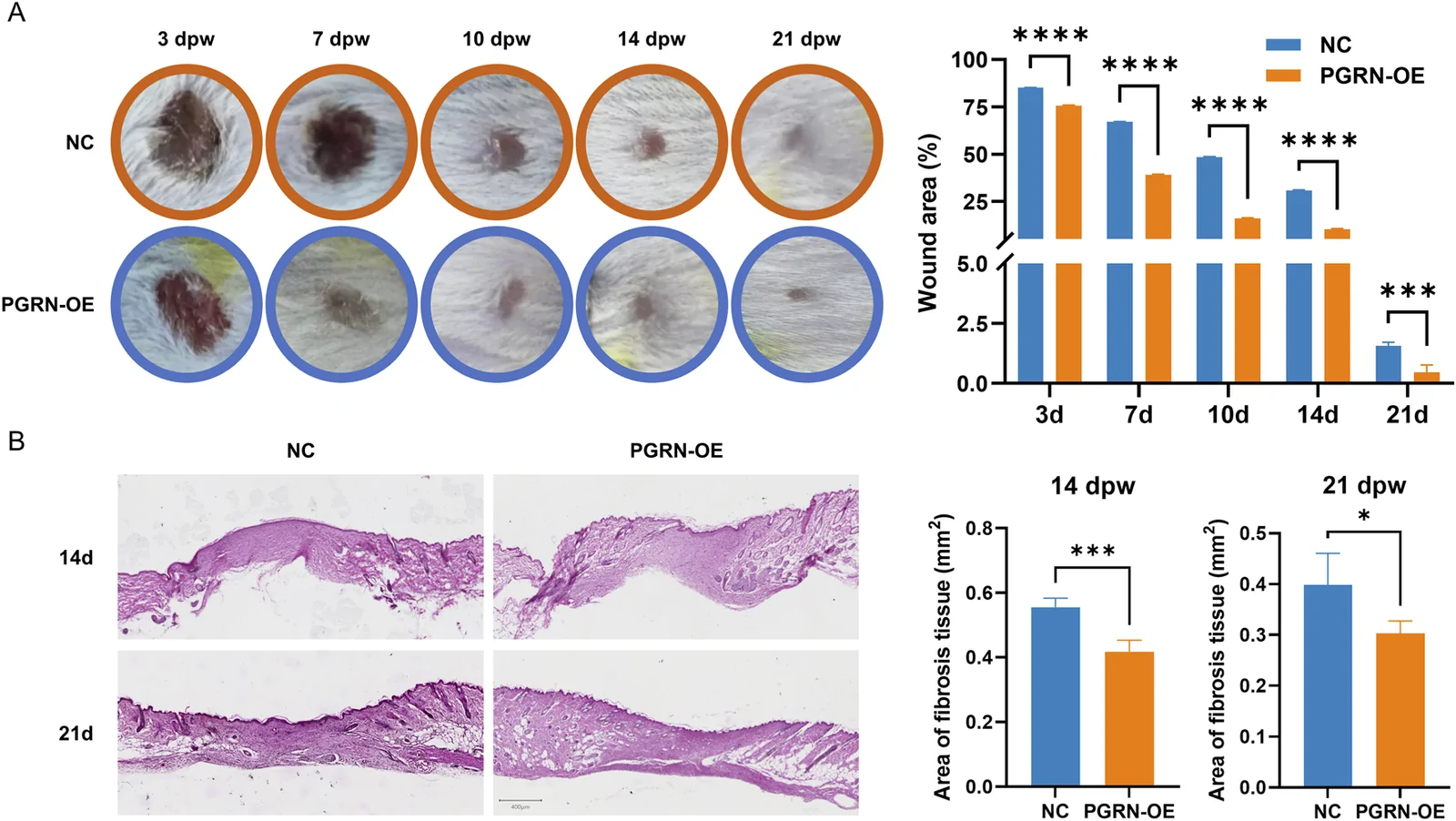
Effect of PGRN on skin wound healing. **(A)** Representative images of mouse skin wounds (left) and statistical analysis of the wound area (right). **(B)** Representative images of H&E staining (left) and statistical analysis of the fibrosis area (right) at 14, and 21 dpw, *P < 0.05, ***P < 0.001, ****P < 0.0001.

### Single-cell RNA-seq reveals the heterogeneity of PGRN-treated mouse skin wounds

To explore the mechanism of PGRN in skin wound healing, we performed scRNA-seq on 7-dpw mouse wounds treated with PGRN-overexpressing lentivirus (PGRN‒OE) or negative control lentivirus (NC) (Figure 2A). After quality control, 22,119 cells were obtained, including 10,672 experimental and 11,447 control cells. The data underwent dimensionality reduction and clustering via uniform manifold approximation and projection (UMAP), revealing 33 distinct cell clusters (Figure 2B). According to the marker genes, we identified 12 fibroblast clusters (clusters 1–4, 7, 8, 13,14, 18, 22, 30, 31), 3 macrophage clusters (clusters 0, 11, 25), 3 epithelial cell clusters (clusters 6, 15, 21), 1 myofibroblast cluster (cluster 9), and so on (Figure 2C; Supplementary Figure S1A). Further proportion analysis revealed that both the cell numbers and relative ratios of these cell clusters varied between the PGRN‒OE and NC groups. The proportions of macrophages, neutrophils, NK cells and myofibroblasts were increased, whereas the proportions of fibroblasts and epithelial cells were decreased in the PGRN‒OE group (Figure 2D; Supplementary Figure S1B).

**FIGURE 2:**
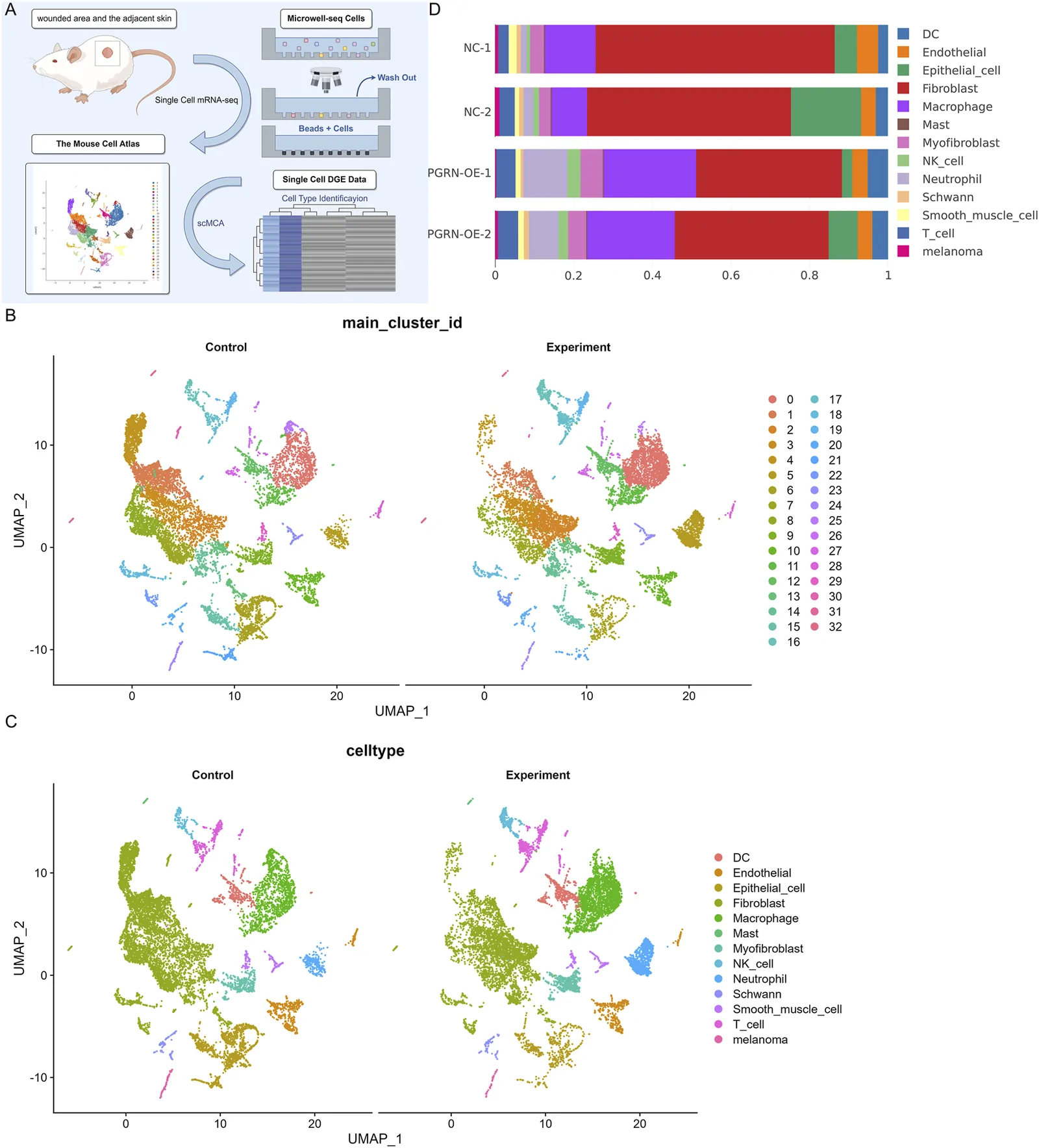
Single-cell RNA sequencing revealed cellular heterogeneity in mouse skin wounds treated with PGRN‒OE or NC lentivirus. **(A)** Experimental workflow for scRNA-seq analysis of full-thickness skin wounds at 7 dpw treated with PGRN‒OE or NC lentivirus (by Figdraw). **(B)** UMAP projection of 22,119 high-quality cells, 10,672 PGRN‒OE (right) vs. 11,447 NC (left), clustered into 33 transcriptionally distinct populations. **(C)** Annotation based on canonical markers identified major cell types: 12 fibroblast clusters, 3 macrophage clusters, 3 epithelial cell clusters, and 1 myofibroblast cluster, PGRN‒OE (right) vs. NC (left). **(D)** Comparative analysis of the cellular composition revealed significant differences between the groups.

### Reclustering of myofibroblasts reveals the impact of PGRN on myofibroblast heterogeneity

Myofibroblasts are critical for skin wound healing because they promote extracellular matrix (ECM) deposition, which contributes to granulation tissue formation and wound closure and enhances tissue resilience and strength (Shook et al., 2018). In this study, the proportion of myofibroblasts increased. The myofibroblasts were reclustered into 6 subclusters via UMAP (Figure 3A), and the top marker genes are shown in Supplementary Figure S2. Compositional analysis revealed that the proportion of the myofibroblast subcluster (M)2 increased in the PGRN‒OE group, whereas that of subcluster M5 decreased; the remaining subclusters were not significantly different (Figure 3B; Supplementary Figure S3A). In terms of marker genes reported in the literature, subcluster M2 tended to be involved in inflammation; subcluster M3 tended to be perivascular, whereas subcluster M4 contributed to perivascular and cell proliferation. All subclusters, except subcluster M5, exhibited contractile characteristics. Subcluster M5 presented low expression of these markers (Supplementary Figure S3B). Subsequent Gene Ontology (GO) and Kyoto Encyclopedia of Genes and Genomes (KEGG) analyses revealed that it is involved in the composition of the ECM (Figure 6A). Differential expression analysis of subclusters M2 and M5 revealed no significant differences (data not shown).

**FIGURE 3:**
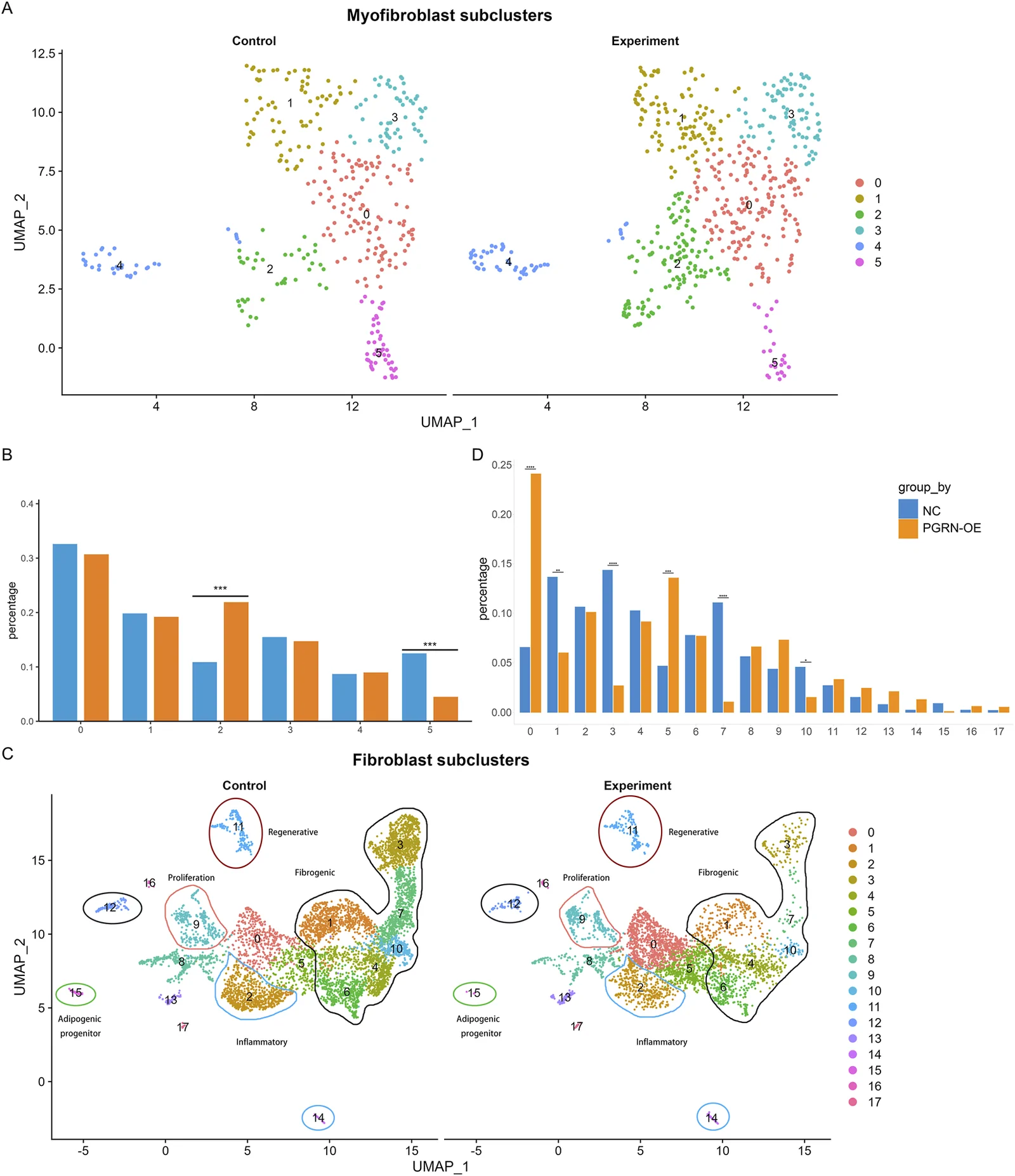
Subclustering of myofibroblasts and fibroblasts revealed PGRN-driven heterogeneity. **(A)** UMAP visualization of myofibroblasts showing six transcriptionally distinct subclusters, PGRN‒OE (right) vs. NC (left). **(B)** Compositional analysis of myofibroblast subclusters, ***P < 0.001. **(C)** UMAP plots of fibroblasts revealed 18 transcriptionally distinct subclusters, PGRN‒OE (right) vs. NC (left). Functional annotation identified fibrotic, regenerative, and other subtypes as indicated. Unidentified subtypes were not circled. **(D)** Compositional analysis of fibroblast subclusters, *P < 0.05, **P < 0.01, ****P < 0.0001.

### Reclustering of fibroblasts reveals the impact of PGRN on fibroblast heterogeneity

Given the close correlation between fibroblasts and skin wound fibrogenesis, we prioritized fibroblasts for further study. The fibroblasts were reclustered into 18 subclusters via UMAP (Figure 3C), and some of the marker genes are shown in Supplementary Figure S4A and B. Compositional analysis revealed that the F0 and F5 subclusters were elevated, whereas the F1, F3, F7, and F10 subclusters were decreased in the PGRN‒OE group, and the remaining subclusters were not significantly different (Figure 3D; Supplementary Figure S3C).

Based on marker genes from previous studies, the fibroblast subclusters were annotated as follows. F1, F3, F4, F6, F7, F10, and F12 were identified as profibrotic fibroblasts, F11 as regenerative fibroblasts, F15 as an adipogenic progenitor, F9 as proliferative fibroblasts, F2 and F14 as inflammatory. The remaining subclusters, F0, F5, F8, F13, F16, and F17, which presented characteristics of both profibrotic and regenerative fibroblasts or neither, were categorized as unidentified (Supplementary Figure S3D). Proportion analysis further revealed that PGRN treatment enriched the proliferative and unidentified subsets while suppressing the profibrotic population (Supplementary Figure S3E). Since we reported that PGRN inhibits fibrogenesis during mouse skin wound healing (Li et al., 2023), elevated F0 and F5 levels should be considered.

### Pseudotime analysis of myofibroblasts and fibroblasts

Pseudotime analysis was applied to arrange the cells according to their transcriptional profiles. Although the overall differentiation trajectory of fibroblasts and myofibroblasts was comparable between PGRN‒OE and NC groups (Figure 4A), PGRN‒OE cells were shifted toward earlier pseudotime (Figure 4B). Three lineages were resolved, lineage 1 originated from early fibroblast subpopulations, while lineages 2 and 3 progressed to later states (Figure 4C). Lineage composition differed markedly between conditions, PGRN‒OE decreased lineage 1 and sharply reduced lineage 2, whereas lineage 3, containing all myofibroblasts, was slightly increased (Figure 4D). At the subcluster level, lineage 1 was dominated by F0, lineage 2 b y F3, and lineage 3 b y F8. F0 occupied the trajectory root and F5 the intermediate region (Figures 4E,F).

**FIGURE 4:**
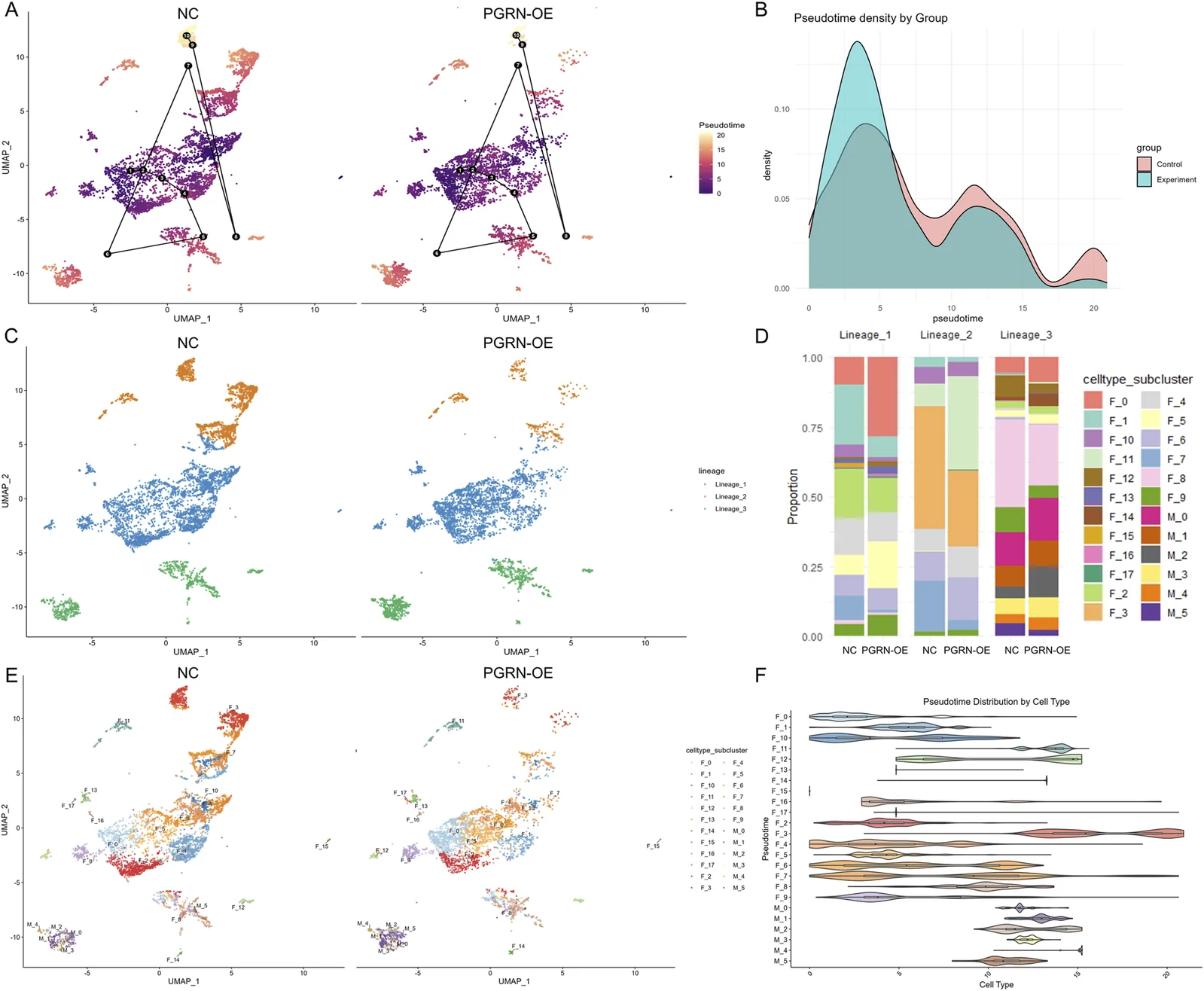
Pseudotime analysis revealed that fibroblast fate trajectories were modulated by PGRN. **(A)** UMAP colored by Monocle 3 pseudotime with the learned trajectory graph overlaid for NC (left) and PGRN–OE (right). Darker tones indicated earlier pseudotime, brighter tones indicated later pseudotime. **(B)** Density distribution of pseudotime values. **(C)** Three lineage trajectories inferred by Monocle3. **(D)** Subcluster composition within each lineage for NC and PGRN–OE. **(E)** UMAP colored by subclusters, fibroblast **(F)** and myofibroblast (M). **(F)** Violin plot showed pseudotime distribution across individual subclusters.

To identify transcriptional programs driving these trajectories, we analyzed pseudotime-dependent genes. In total, 2,388 genes showed significant autocorrelation along pseudotime. Highly variable genes were enriched in ECM remodeling (Tnxb, Mfap5, Cthrc1, Tinagl1, Clec3b), myofibroblast contractility (Myh11, Lrrc15), and metabolic/adipose signatures (Adipoq, Retn, Higd1b). We also analyzed branch-dependent genes across the three lineages and identified the top 50, including Mfap5, Cthrc1, Postn, Fn1, and Lox/Loxl1. Lineage 1 was enriched for ECM synthesis/remodeling, lineage 2 for contractile programs, and lineage 3 for vascular/ECM maturation, suggesting that PGRN rerouted fibroblasts toward a vascular/pericyte-associated and remodeling state (Supplementary Figure S5).

### Potential ligand–receptor interaction analyses in fibroblasts and myofibroblasts

To elucidate the intercellular communication between fibroblast and myofibroblast subpopulations, an analysis was conducted via the CellPhoneDB. The most vigorous cell–cell interactions were observed in subclusters F5 and F13, whereas F8 and M5 presented the fewest interactions (Figure 5A). A comparison between the PGRN‒OE group and the NC group revealed a reduction in ligand‒receptor interactions involving Tenascin-C (Tnc)_Syndecan 1 (Sdc1) and Col6a5_Cd44 within the PGRN‒OE group and an increase in interactions of Col1a1_Cd44, Tenascin-X (Tnxb)_Sdc1, and C-X‒C Motif Chemokine Ligand 12 (Cxcl12) _Atypical Chemokine Receptor 3 (Ackr3) (Figure 5B; Supplementary Figure S6). We subsequently examined well-established ligand‒receptor pairs relevant to fibrosis and wound healing. The most dynamic interactions involved transforming growth factor beta 1 (TGFβ) signaling (Tgfb2/Tgfb3_Tgfbr1/Tgfbr2) and vascular endothelial growth factor (VEGF) signaling (Vegfa/Vegfb_Flt1). In the PGRN‒OE group, pathways that were significantly upregulated included VEGF signaling (Vegfa_Kdr) and TGFb3 signaling (Tgfb3_Tgfbr1/Tgfbr2). In contrast, pathways that were notably downregulated within the PGRN‒OE group included insulin-like growth factor 2 (IGF2) signaling (Igf2_Igf1r) (Figure 5C). The TGFβ pathway across cell subclusters is illustrated in Figure 5D.

**FIGURE 5:**
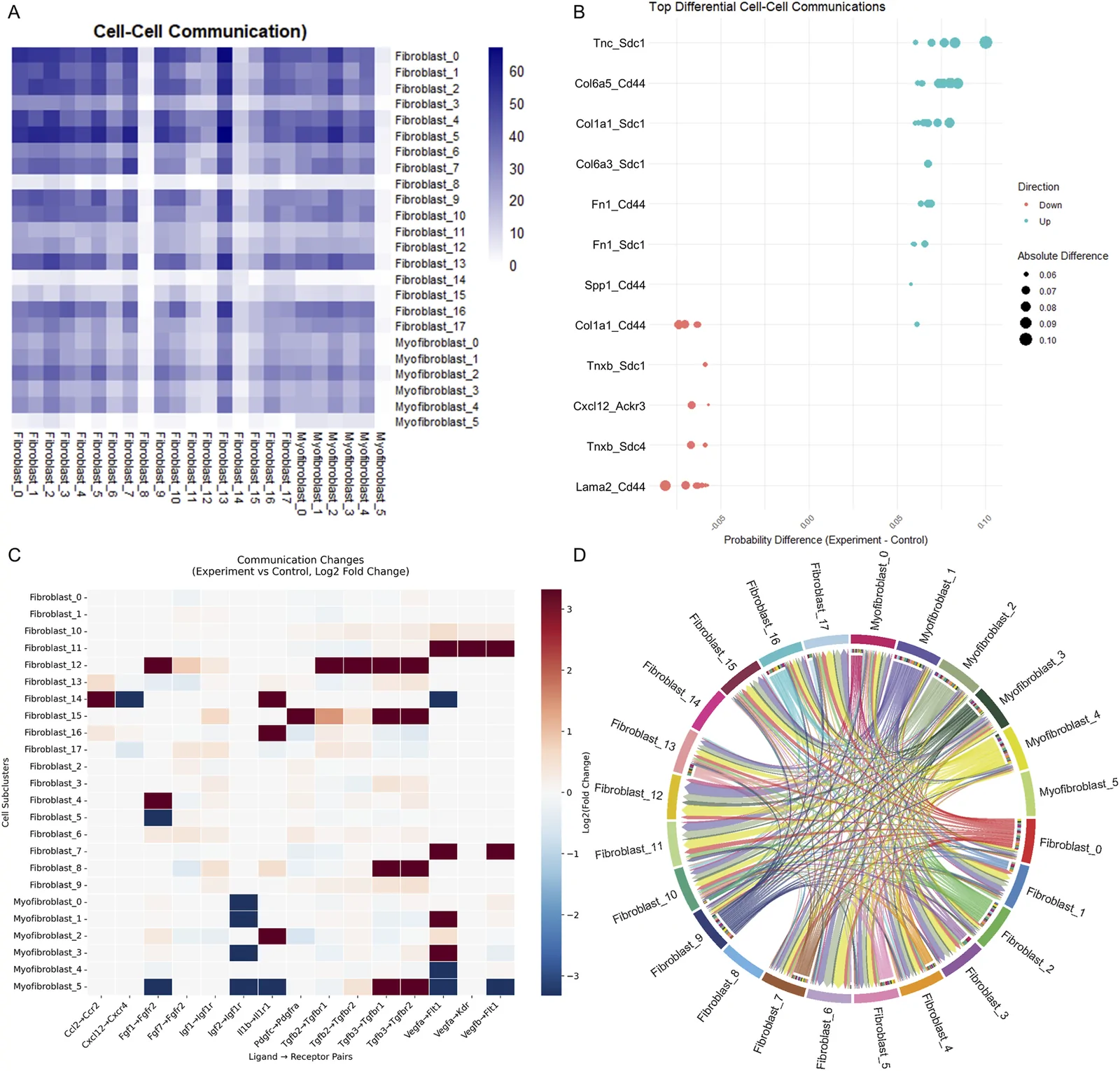
Evaluation of the potential for cell–cell communication between fibroblast and myofibroblast subclusters. **(A)** Heatmap representing the strength of cell–cell communication. **(B)** Top differential cell–cell interactions between the PGRN–OE and NC groups. **(C)** Differences in cell–cell communication related to wound healing and fibrosis between the PGRN–OE and NC groups. **(D)** Differential cell–cell communication related to the TGF signaling pathway.

### Fibroblast function exploration of subclusters F0 and F5

We further analyzed the highly variable genes in the fibroblasts of subclusters F0 and F5 (Supplementary Figure S4A and B). Gene Ontology (GO) enrichment and KEGG pathway analyses revealed that the highly variable genes in these two subclusters were significantly enriched in pathways related to the collagen-containing extracellular matrix, cell-substrate adhesion, the extracellular matrix structural constituents conferring tensile strength, epithelial cell proliferation, and the regulation of angiogenesis. Notably, subcluster F0 was enriched in the protein secretion pathway, whereas subcluster F5 focused particularly on the fatty acid metabolic process and the Pi3K‒Akt signaling pathway (Figure 6B). Given the crucial role of angiogenesis in wound healing, we assessed blood flow in newly formed tissue postinjury via a laser speckle imager. The results indicated that the PGRN‒OE group had notably greater blood flow than did the NC group at 7 dpw, with no significant differences at 14 and 21 dpw (Figure 6C), indicating that PGRN promotes angiogenesis in the early stages of proliferation. Differential expression analysis was conducted on subclusters F0 and F5. The analysis revealed that, in the PGRN‒OE group, Col6a1, Col6a2, Col6a5, Ccl11, *etc.*, were upregulated in both subclusters. Genes such as Acta2 and Lgr5 were downregulated in subcluster F0, whereas genes such as Zbtb16 and Clec3b were downregulated in subcluster F5 (Supplementary Figure S4C and D).

**FIGURE 6:**
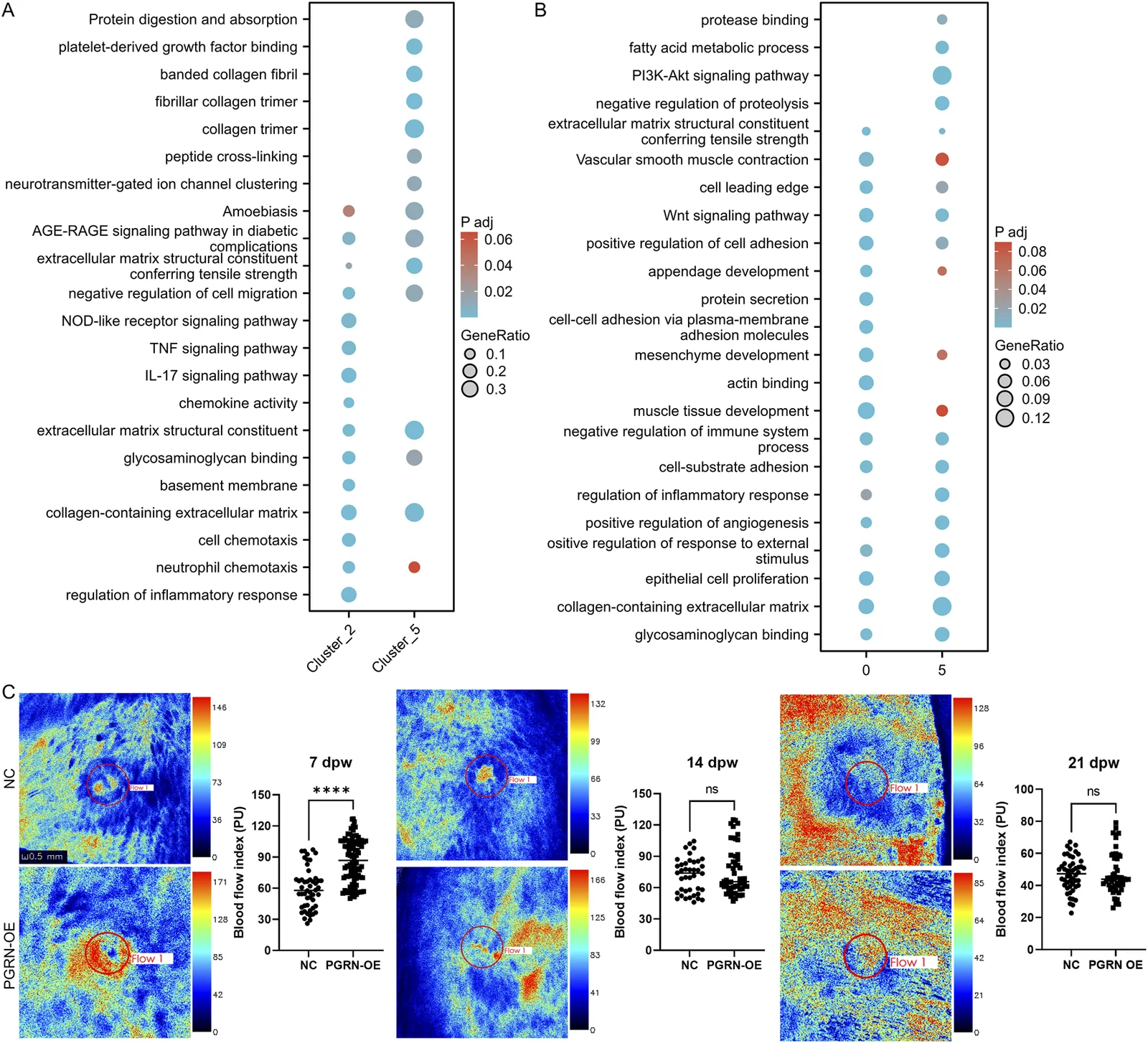
Functional annotation of subclusters F0 and F5. **(A)** GO and KEGG analyses of subclusters M2 and M5. **(B)** GO/KEGG enrichment analysis of subclusters F0 and F5. **(C)** Laser speckle imaging revealed that, compared with NC wounds, PGRN–OE wounds presented a greater perfusion index in neotissue, indicating the angiogenic function of subclusters F0 and F5 (left) and the statistical analysis of the WBI at 7, 14, and 21 dPw (right), ****P < 0.0001.

### PGRN promoted Crabp1+/Prss35+/Fabp5+/Acta2+ fibroblasts

Subcluster F5 revealed only 5 highly variable genes: secreted frizzled-related protein 2 (Sfrp2), periostin (Postn), pleiotrophin (Ptn), insulin-like growth factor binding protein 4 (Igfbp4) and matrix metallopeptidase 3 (Mmp3). Interestingly, none of these genes were unique to subcluster F5 (Supplementary Figure S4A). Subcluster F0 revealed 41 biomarkers, including cellular retinoic acid binding protein 1 (Crabp1), serine protease 35 (Prss35), fatty acid binding protein 5 (Fabp5), actin alpha 2, smooth muscle (Acta2), and collagen type VII alpha 1 chain (Col7a1) (Figure 7A; Supplementary Figure S4B). Thus, we chose these genes to identify subcluster F0. Analysis of the GSE178758 spatial transcriptomic data revealed the spatial locations of Crabp1+/Prss35+/Fabp5+/Acta2+ cells in the upper layer of the neoderm at 7 dpw (Figure 7B). Multiple immunohistochemical (mIHC) staining was performed to characterize subcluster F0 cells. The results revealed that both Fabp5+/Acta2+ and Crabp1+/Prss35+ cells were localized in the upper layer of the neodermis in the wound. Furthermore, Crabp1+/Prss35+ cells were also present in the hair follicles (Figure 7C). Immunoblot analysis revealed that PGRN‒OE increased PRSS35 expression, whereas CRABP1 levels were not significantly different at 7 dpw (Figure 7D).

**FIGURE 7:**
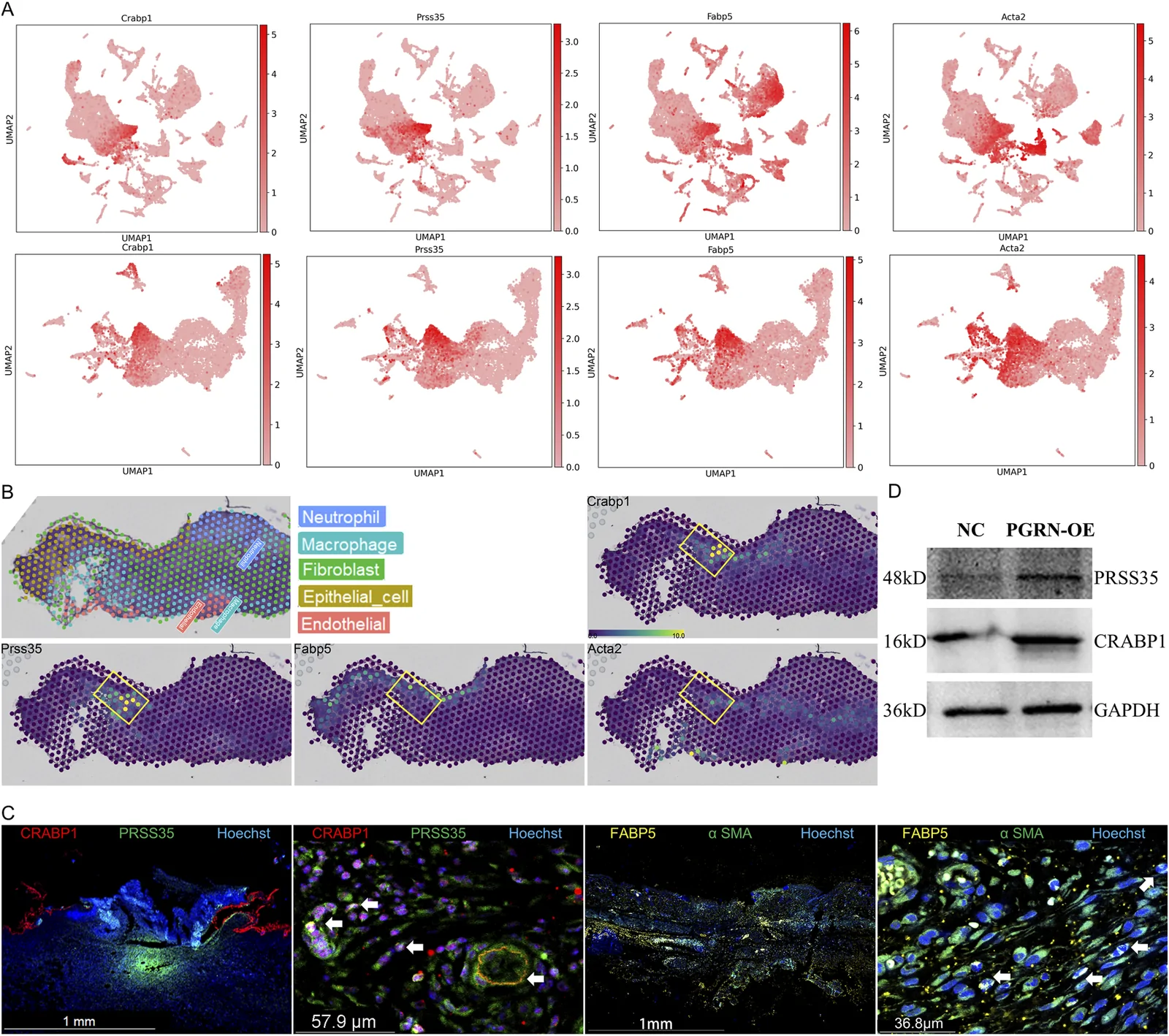
Subcluster F0 is characterized by Crabp1, Prss35, Fabp5, and Acta2 and is involved in regenerative wound healing. **(A)** UMAP plots showing exclusive enrichment of Crabp1, Prss35, Fabp5, and Acta2 in subcluster F0 (upper: pantissue; lower: fibroblasts). **(B)** Spatial transcriptomics dataset GSE178758 showing the locations of Crabp1, Prss35, Fabp5, and Acta2 at 7 dpw. **(C)** Representative images of Crabp1+/Prss35+ and Fabp5+/Acta2+ double-positive fibroblasts in granulation tissue (arrow). **(D)** Representative images of immunoblots for Crabp1 and Prss35.

## Discussion

Wound healing is a complex biological process that requires coordination among different cell types, such as fibroblasts, macrophages, and epithelial cells (Talbott et al., 2022). Previous reports have shown that PGRN affects fibroblast behavior and regulates fibrosis and tissue regeneration (Yang et al., 2024). Our previous studies demonstrate that PGRN attenuates fibrosis during mouse skin wound healing, with macrophage-derived extracellular vesicles serving as a major source of PGRN (Li et al., 2023; Huang et al., 2026). Understanding the diversity and functions of fibroblasts in response to PGRN treatment is essential for developing targeted therapies to improve wound healing outcomes.

In this study, we focused on the role of PGRN in skin wound healing, particularly its potential to modulate fibrogenesis during the healing process. By using scRNA-seq, we investigated how PGRN influences the heterogeneity of cell populations within the wound environment. Our findings revealed that PGRN treatment significantly altered the composition of key cell types in the wound environment, increasing the number of macrophages, neutrophils, natural killer cells, and myofibroblasts while decreasing the number of fibroblasts and epithelial cells at 7 dpw.

Interestingly, the PGRN‒OE group presented increased myofibroblast populations. Moreover, pseudotime analyses indicated that PGRN promoted lineage 3, which included all the myofibroblast subclusters. This finding implies that PGRN may play a pivotal role in myofibroblast differentiation. Myofibroblasts are traditionally viewed as collagen-producing cells. However, their transdifferentiation does not always correlate with increased deposition of collagen in the ECM. Recent studies highlight the heterogeneity of myofibroblast populations, suggesting that they are not a uniform cell type but rather consist of diverse subpopulations with distinct phenotypes and functions (Shook et al., 2018; Gao et al., 2024). In this study, we recustered myofibroblasts into six distinct subclusters, focusing on their roles in contraction, inflammation, perivascular activity, and proliferation. The increase in subcluster M2 and decrease in subcluster M5 within the PGRN‒OE group suggested a shift toward a functional myofibroblast phenotype that may enhance inflammation and prevent excessive fibrosis.

The investigation of fibroblast heterogeneity revealed 18 distinct subclusters. These subclusters exhibited notable variations in their expression patterns and interactions with myofibroblast subclusters in response to PGRN. PGRN suppressed Tnc_Sdc1 but enhanced Tnxb_Sdc1 interactions. Since Tnc elevates TGFβ levels in dermal fibroblasts (Jinnin et al., 2004) and Tnxb knockout amplifies TGFβ signaling in vascular smooth muscle cells (Liang et al., 2024), this modulation likely inhibits profibrotic signaling. Additionally, PGRN enhances Cxcl12_Ackr3 signaling, which may be associated with ECM remodeling and skin regeneration (Cuesta-Margolles et al., 2025). With respect to the interactions related to wound healing and fibrosis, communication between TGFβ3 and its receptors TGFBR1 and TGFBR2 was significantly increased in the PGRN‒OE group. Lichtman, M K et al. reviewed that TGFβ3 effectively reduces scarring (Lichtman et al., 2016); therefore, the upregulation of TGFβ3 signaling in the PGRN‒OE group may underlie the inhibitory effect of PGRN on fibrogenesis.

Notably, a significant decrease in fibroblast populations was observed in the PGRN‒OE group. The increase in unidentified fibroblast subtypes (F0 and F5) in the PGRN‒OE group, along with the suppression of profibrotic fibroblasts, suggested that PGRN may modulate fibroblast behavior to optimize therapeutic strategies for wound healing. The pathways enriched in subclusters F0 and F5 were related to extracellular matrix remodeling and angiogenesis, as well as other biological processes. Angiogenesis, which is essential for effective wound healing, is crucial for the development of granulation tissue and improving healing outcomes (Ruehle et al., 2020). Recent studies have emphasized the importance of various molecules and cells that regulate angiogenesis in wound healing. Austin P Veith et al. reviewed the therapeutic strategies that enhance angiogenesis in wound healing, with a focus on the roles of growth factors, nongrowth factor proteins, microRNAs, and stem cells (Veith et al., 2019).

VEGF signaling is a key regulator of vasculogenesis and angiogenesis. These processes are essential for vascular systems, tissue regeneration, and the preservation of homeostasis (Lee et al., 2025). The identification of crucial ligand‒receptor interactions relevant to fibrosis and wound healing revealed that PGRN‒OE enhanced VEGF signaling and thus strengthened the angiogenic effects of PGRN. Additionally, our study revealed that, compared with the NC group, the PGRN‒OE group presented markedly increased blood flow during the early proliferation phase. These findings indicated that PGRN promoted angiogenesis during wound healing. Fibroblast subclusters F0 and F5 play key roles in this process.

Given that the highly variable genes in subcluster F5 appeared in nearly all the fibroblast subclusters, we further focused on mapping the cells of subcluster F0, which were characterized by Crabp1, Prss35, Fabp5, and Acta2. The spatial transcriptomic data, along with mIHC staining, indicated the presence of Fabp5+/Acta2+ and Crabp1+/Prss35+ cells located in the upper layer of the neodermis at the wound site. This finding supports the results of a previous study (Abbasi et al., 2020). In the regenerative wound environment, fibroblasts expressing Crabp1, located in the upper wound, exhibit a gene signature similar to that of papillary fibroblasts. Both upper wound fibroblasts and papillary fibroblasts are essential for developing and maintaining hair follicles (Phan et al., 2021). Hair follicles are crucial for skin wound healing because they provide structural support and stem cells for tissue regeneration (Ji et al., 2021). Skin-derived precursors are capable of regenerating the dermal sheath and dermal papilla cells during each growth cycle, thereby reconstituting hair follicles (Zhang et al., 2024) During wound healing, dermal sheath cells around hair follicles differentiate into fibroblasts. These fibroblasts contribute to angiogenesis and recruit other cell types essential for effective wound healing (Tao et al., 2019; Ji et al., 2021). Our study revealed highly variable genes in subcluster F0 that are markers of both dermal papilla and dermal sheath fibroblasts, and we also detected Crabp1+/Prss35+ cells in hair follicles. These findings led us to propose that subcluster F0 was associated with hair follicles and contributed to effective wound regeneration. This may be one of the mechanisms by which PGRN affects wound healing.

This study has several limitations that should be noted. The lack of *in vivo* functional validation restricts our ability to fully confirm the findings from single-cell RNA sequencing. Our study was limited to young adult male BALB/c mice. Since both age and sex are known to influence wound repair (Blair et al., 2020; Gilliver and Ashcroft, 2007; Plum et al., 2025; Santiago-de-la-Cruz et al., 2026), future investigations should examine the role of PGRN in adult or aged mice, as well as in female mice. Although BALB/c mice are commonly used in skin wound healing studies, our findings may not be generalizable to other strains or species, underscoring the need for validation in diverse models. In addition, single-cell analyses were anchored at day 7, with supporting observations at days 14 and 21. However, these timepoints may not capture the full dynamics and all phenotypes of the wound healing process, studies covering a broader range of timepoints are needed. Given that nerves play an important role in skin fibrosis (Tam et al., 2026), exploring the effects of PGRN on nerve endings represents a promising direction for future research.

## Conclusion

In summary, our study explored the role of PGRN in skin wound healing, emphasizing the changes in cellular composition and fibroblast/myofibroblast heterogeneity caused by PGRN overexpression. PGRN was shown to promote Crabp1+/Prss35+/Fabp5+/Acta2+ fibroblasts, which are associated with hair follicles and contribute to effective wound regeneration. This study provides a potential therapeutic strategy for improving wound healing and reducing fibrosis.

## Data Availability

The datasets presented in this study can be found in online repositories. The names of the repository/repositories and accession number(s) can be found below: https://www.ncbi.nlm.nih.gov/, PRJNA1332166.
