# Supplementary material for: Single-cell RNA-seq analysis reveals the role of PGRN in modulating fibroblast heterogeneity and enhancing wound healing
Source: Front Bioeng Biotechnol. 2026 Oct 13;14:1930259. doi: 10.3389/fbioe.2026.1930259 (PMC13710927; doi:10.3389/fbioe.2026.1930259)
Supplement: Supplementary file 1 [file Supplementaryfile1.docx]

Figure S1 (A) Canonical markers of mouse skin. (B) Comparative analysis of the cellular composition revealed significant differences between the groups (**P*<0.05, *****P*<0.0001).

Figure S2. Subcluster-specific highly variable genes of myofibroblast subclusters

(A) Highly variable genes in subcluster M2. (B) Highly variable genes in subcluster M5.

Figure S3. (A) Compositional analysis of myofibroblast subclusters. (B) Functional annotation of biological processes in myofibroblast subclusters. (C) Compositional analysis of fibroblast subclusters. (D) Functional annotation of biological processes in fibroblast subclusters. (E) Comparative analysis of the cellular composition revealed significant differences between the groups (****P*<0.001).

Figure S4. Highly variable genes specific to fibroblast subclusters

(A) Highly variable genes of subcluster F5. (B) Highly variable genes of subcluster F0. (C) Volcano plot showing DEGs in subcluster F0. (D) Volcano plot showing differentially expressed genes in subcluster F5.

Figure S5. Heatmap displaying the top pseudotime varying (left) and branch-dependent genes (right).

Figure S6. The top 10 unique ligand–receptor pairs of fibroblasts and myofibroblasts.
